# Reativação da Doença de Chagas após Transplante Cardíaco: Importância de Novos Preditores

**DOI:** 10.36660/abc.20240284

**Published:** 2024-07-01

**Authors:** Roberto M. Saraiva, Andréa R. Costa

**Affiliations:** 1 Instituto Nacional de Infectologia Evandro Chagas Fundação Oswaldo Cruz Rio de Janeiro RJ Brasil Instituto Nacional de Infectologia Evandro Chagas – Fundação Oswaldo Cruz, Rio de Janeiro, RJ – Brasil

**Keywords:** Palavras-chave: Doença de Chagas, Infecção Latente, Fatores de Risco

A doença de Chagas (DC) crônica afeta cerca de 3,7 milhões de brasileiros segundo estimativa mais recente.^1^ Como cerca de 30-40% desta população apresenta a forma cardíaca, não surpreende que a DC seja a terceira etiologia mais frequente entre os pacientes submetidos a transplante cardíaco no Brasil.^2^ Como a terapia imunossupressora de indução e/ou manutenção acarreta risco de reativação da DC (RDC),^3^ a segurança do transplante cardíaco pode ser questionada na DC. Entretanto, a experiência no Brasil estabeleceu o transplante cardíaco como a principal alternativa de tratamento para pacientes com DC e insuficiência cardíaca terminal.^2^ Na verdade, a sobrevida pós-transplante de pacientes com DC no Brasil é de 76%, 71% e 46% após 6 meses, cinco e 10 anos, respectivamente, e melhor do que a sobrevida de receptores de transplante cardíaco com doença isquêmica ou cardiomiopatias idiopáticas.^4,5^

A incidência de RDC após transplante cardíaco varia de 19,6% a 90%.^3^ A RDC pode induzir sintomas de DC aguda (febre, anemia, icterícia), miocardite, paniculite, meningoencefalite e abscesso cerebral. A miocardite é a complicação mais frequente e pode apresentar sintomas graves compatíveis com insuficiência cardíaca, arritmia cardíaca e até choque cardiogênico.^6^ Felizmente, a RDC adequadamente diagnosticada e tratada resulta em mortalidade inferior a 1%.^6^

No entanto, os episódios de rejeição também podem apresentar resultados semelhantes e um diagnóstico equívoco de rejeição em vez de RDC pode levar a consequências nefastas se for seguida uma intensificação do regime imunossupressor.^6^ O diagnóstico de RDC é classicamente baseado na presença de achados clínicos sugestivos e evidências do parasita no sangue, líquor, medula óssea ou tecidos.^3,4^ Portanto, protocolos para monitoramento de RDC foram desenvolvidos e hoje incluem PCR para T. cruzi em sangue e biópsias endomiocárdicas, que são mais sensíveis que os métodos parasitológicos padrão, como a observação direta do parasita em um esfregaço de sangue ou numa biópsia endomiocárdica ou uma hemocultura positiva. O objetivo é o diagnóstico precoce da RDC, iniciando o tratamento tripanocida antes do aparecimento de sintomas graves e danos ao coração transplantado. É importante ressaltar que uma PCR sanguínea positiva para T. cruzi precede o aparecimento de sinais clínicos de RDC com considerável sensibilidade e especificidade.^7^ Além disso, uma PCR sanguínea negativa para T. cruzi exclui a RDC.^8^ Os resultados da PCR são fundamentais para orientar decisões terapêuticas entre medicamentos tripanocidas ou alterações nos regimes de imunossupressão.^8,9^ Alguns autores consideram que o diagnóstico de RDC deve ser redefinido como presente mesmo na ausência de sintomas clínicos evidentes, desde que um aumento na parasitemia possa ser detectado por técnicas parasitológicas diretas ou por PCR.^6^

Além de um diagnóstico correto de RDC, o reconhecimento dos fatores de risco para tal evento é importante. Eles incluem, o número de episódios de rejeição, presença de malignidade, grau de imunossupressão, doenças autoimunes, infecção por HIV e outros estados de imunossupressão.^10^ Portanto, as estratégias para prevenir a reativação induzida pela rejeição geralmente incluem o uso das doses mais baixas de terapia imunossupressora de vários medicamentos.^4,6^

Devido à importância da RDC, a identificação de fatores de risco que permitam diagnóstico e tratamento precoces é fundamental. Nesta edição dos Arquivos Brasileiros de Cardiologia, Wolf et al.^11^ descreveram que a contagem absoluta de linfócitos abaixo de 550/mm^3^ durante as primeiras 2 semanas após o transplante cardíaco foi um preditor de uma subsequente PCR sanguínea positiva para T. cruzi.^11^ Na verdade, como a terapia imunossupressora de indução induz linfodepleção e a resposta imune de células T CD4+ e CD8+ contra T. cruzi, é relevante tanto para o controle do parasita quanto para a patogênese da doença,^12^ uma baixa contagem de linfócitos pode ocorrer antes da RDC. Este fator de risco precoce e prontamente disponível para uma PCR sanguíneo positiva para T. cruzi pode se tornar muito útil no acompanhamento após transplante cardíaco em receptores com DC. Uma contagem baixa de linfócitos pode levar a uma avaliação PCR mais precoce ou a uma mudança no tratamento imunossupressor. Além disso, uma contagem elevada de linfócitos poderia adiar uma avaliação de PCR, o que pode ser útil para serviços com acesso mais difícil às técnicas de PCR. Outra possibilidade é o tratamento tripanocida preventivo baseado na baixa contagem de linfócitos. Todas estas possíveis aplicações clínicas para a contagem de linfócitos durante as primeiras duas semanas após um transplante cardíaco devem ser confirmadas por ensaios clínicos devidamente concebidos.
