# Supplementary material for: Reativação da Doença de Chagas após Transplante Cardíaco: Importância de Novos Preditores
Source: Arq Bras Cardiol. 2024 Jul 1;121(6):e20240284. [Article in Portuguese] doi: 10.36660/abc.20240284 (PMC12080714; doi:10.36660/abc.20240284)
Supplement: Translation

# Translation: Chagas Disease Reactivation after Heart Transplant: Importance of New Predictors

**DOI:** 10.36660/abc.20240284i

**Authors:** Roberto M. Saraiva, Andréa R. Costa

**Affiliations:** 1 Brazil Instituto Nacional de Infectologia Evandro Chagas – Fundação Oswaldo Cruz, Rio de Janeiro, RJ – Brazil

**Keywords:** Chagas Disease, Latent Infection, Risk Factors

Chronic Chagas disease (CD) affects around 3.7 million Brazilians according to the most recent estimative.^1^ As around 30-40% of this population present with the cardiac form, it comes as no surprise that CD is the third most frequent etiology among patients undergoing heart transplant in Brazil.^2^ As induction and/or maintenance immunosuppressive therapy carry the risk of CD reactivation (CDR),^3^ heart transplant safety could be questioned in CD. However, the experience in Brazil established heart transplants as the leading alternative treatment for CD patients with end-stage heart failure.^2^ In fact, the post-transplant survival of patients with CD in Brazil is 76%, 71%, and, 46% after 6 months, five, and 10 years, respectively, and better than the survival of heart transplant recipients with either ischemic or idiopathic cardiomyopathies.^4,5^

CDR incidence after heart transplant varies from 19.6% to 90%.^3^ CDR can induce symptoms of acute CD (fever, anemia, jaundice), myocarditis, panniculitis, meningoencephalitis, and brain abscess. Myocarditis is the most frequent complication and may present severe symptoms compatible with heart failure, cardiac arrhythmia, and even cardiogenic shock.^6^ Fortunately, CDR properly diagnosed and treated results in less than 1% mortality.^6^

However, rejection episodes may also present with similar findings and an equivocal diagnosis of rejection instead of CDR can lead to ominous consequences if an intensification of the immunosuppressive regimen is ensued.^6^ CDR diagnosis is classically based on the presence of suggestive clinical findings and evidence of the parasite in blood, liquor, bone marrow, or tissues.^3,4^ Therefore, protocols for monitoring CDR were developed and nowadays include PCR for *T. cruzi* in blood and endomyocardial biopsies, which are more sensitive than standard parasitological methods in such as direct observation of the parasite in a blood smear or an endomyocardial biopsy or a positive blood culture. The objective is an early diagnosis of CDR prompting trypanocidal treatment before the onset of severe symptoms and damage to the transplanted heart. Importantly, a positive PCR for *T. cruzi* in blood precedes the appearance of clinical signs of CDR with considerable sensitivity and specificity.^7^ Furthermore, a negative blood PCR for *T. cruzi* rules out CDR.^8^ PCR results are fundamental to guide therapeutic decisions between trypanocidal drugs or changes in immunosuppression regimens.^8,9^ Some authors consider that CDR diagnosis should be redefined as present even in the absence of evident clinical symptoms as long as an increase in parasitemia can be detected either by direct parasitological techniques or by PCR.^6^

Beyond a correct diagnosis of CDR, the recognition of the risk factors for such an event is important. Those are listed as follows, the number of rejection episodes, presence of malignancy, immunosuppression grade, autoimmune diseases, HIV infection, and other immunosuppression status.^10^ Therefore, strategies to prevent rejection-induced reactivation generally include the use of the lowest immunosuppressive therapy doses of several drugs.^4,6^

Due to the importance of CDR, the identification of risk factors that allow an early diagnosis and treatment is fundamental. In this issue of the *Arquivos Brasileiros de Cardiologia,* Wolf et al.^11^ described that absolute lymphocyte count under 550/mm^3^ during the first 2 weeks after heart transplant was a predictor of a subsequent positive blood PCR for *T. cruzi.*^11^ In fact, as induction immunosuppressive therapy induces lymphodepletion and CD4^+^ and CD8^+^ T cell immune response against *T. cruzi* is relevant for both parasite control and disease pathogenesis,^12^ a low lymphocyte count can occur before CDR. This early and readily available risk factor for a positive blood PCR for *T. cruzi* can become very useful for the follow-up after a heart transplant in CD recipients. A low lymphocyte count can prompt an earlier PCR evaluation or a change in immunosuppressive treatment. Also, a high lymphocyte count could postpone a PCR evaluation, which can be useful for services with more difficult access to PCR techniques. Another possibility is preemptive trypanocidal treatment based on low lymphocyte count. All these possible clinical applications for lymphocyte count during the first two weeks after a heart transplant should be confirmed by properly designed clinical trials.
